# Why does invasive brain stimulation sometimes improve memory and sometimes impair it?

**DOI:** 10.1371/journal.pbio.3002894

**Published:** 2024-10-25

**Authors:** Uma R. Mohan, Joshua Jacobs

**Affiliations:** 1 Surgical Neurology Branch, NINDS, National Institutes of Health, Bethesda, Maryland, United States of America; 2 Department of Biomedical Engineering, Columbia University, New York City, New York, United States of America; 3 Department of Neurological Surgery, Columbia University, New York City, New York, United States of America

## Abstract

Invasive brain stimulation is used to treat individuals with episodic memory loss; however, studies to date report both enhancement and impairment of memory. This Essay discusses the sources of this variability, and suggests a path towards developing customized stimulation protocols for more consistent memory enhancement.

## Author summary

Episodic memory is a critical aspect of everyday life that allows us to recall events, make decisions, and navigate our environments. It is estimated that 1 in 25 Americans suffer from episodic memory loss from Alzheimer’s disease, dementia, or following a traumatic brain injury. Clinicians and researchers have recently tried to help supplement or repair memory in individuals with episodic memory disorders using electrical brain stimulation. However, although there is evidence of invasive brain stimulation improving memory, studies also show that it can have variable effects, and sometimes cause memory impairment. In this Essay, we investigate these differences and try to explain this variability. We propose that key aspects of the memory network are heterogeneous across individuals, requiring customized stimulation protocols to create successful brain stimulation systems for memory enhancement. We provide a plan for applying brain stimulation while overcoming this variability by using closed-loop stimulation protocols that are customized according to a person’s own anatomical structure, functional connectivity, and memory-related electrophysiological patterns.

## Introduction

Direct electrical brain stimulation is an effective treatment for a number of neurological and behavioral disorders, including Parkinson’s disease, essential tremor, dystonia, and epileptic seizures [1–9]. Building on this success, in the past decades researchers have expanded the scope of invasive brain stimulation, using it to help patients with a much broader range of neuropsychiatric and cognitive disorders, including major depressive disorder [10,11], obsessive compulsive disorder [12], anorexia nervosa [13], addiction [14,15], schizophrenia [16,17], and memory disorders such as Alzheimer’s disease [18,19]. However, unlike the earlier success of deep brain stimulation for motor disorders, these efforts at using brain stimulation to treat neuropsychiatric and cognitive disorders have produced inconsistent effects [2,3,20,21]. But why is this the case?

**Fig 1:**
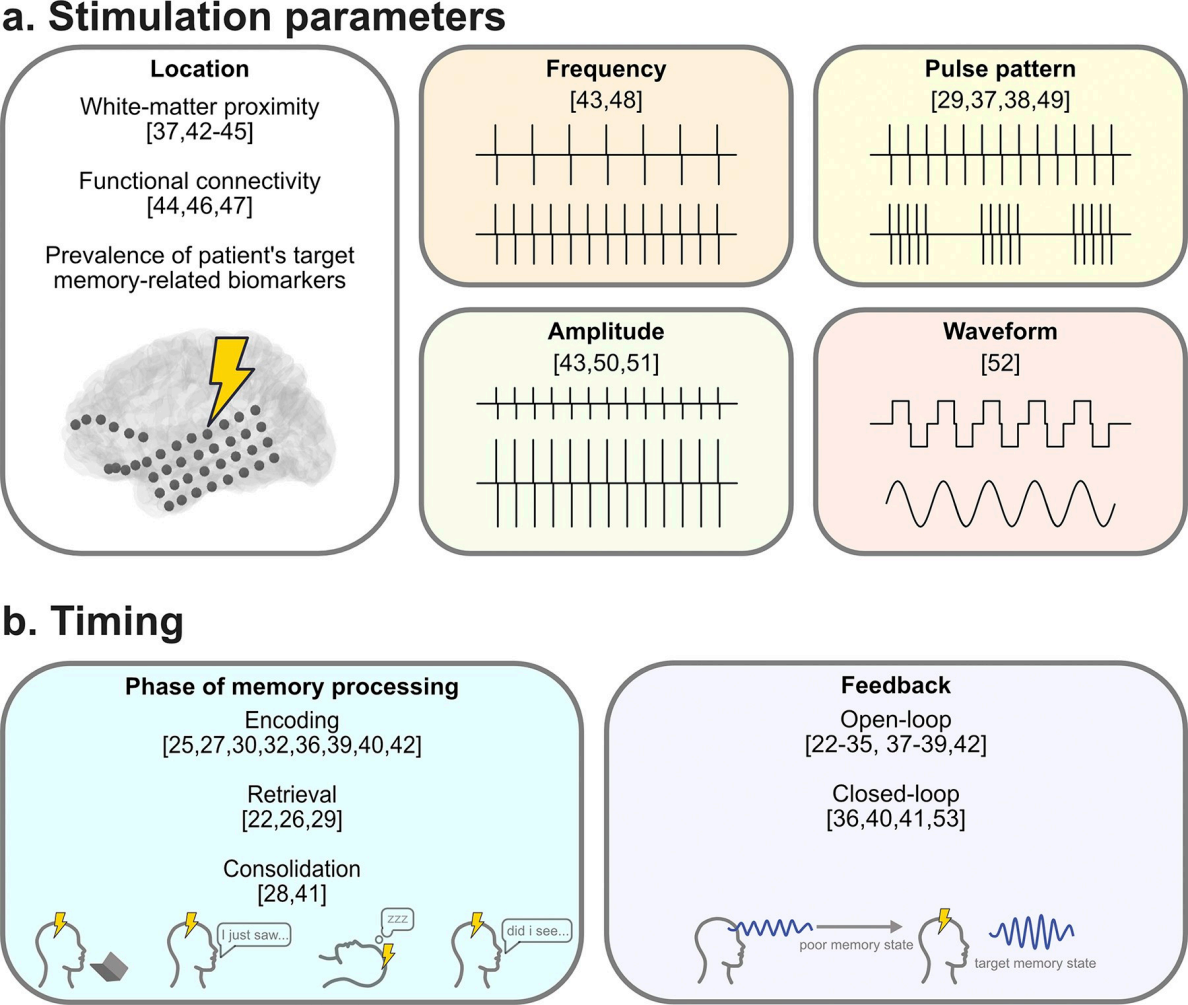
Stimulation parameter, location, and timing considerations. (**a**) Different stimulation locations, frequencies, amplitudes, durations, and pulse widths have been shown to have varying effects on neural activity and in turn on memory function. When developing a stimulation protocol designed to modulate a specific memory-related neural signal, it is critical to first determine how the combination of these parameters will alter electrophysiology [29,37,38,42–52]. (**b**) Separate from the parameters that determine how current is delivered, the timing, or state in which an individual is in, impacts the effect of stimulation on memory. These can be considered in 2 different ways: timing with respect to the targeted memory process determined by the structure of the memory task [22,25–30,32,36,39–42], and timing from moment-to-moment, i.e., whether an individual’s neural signals show that they are in a good or bad memory processing state [36,53].

Previous studies on the use of brain stimulation for memory enhancement generally used fixed stimulation protocols across individuals. These studies produced wide-ranging outcomes, with some reporting impaired memory performance from stimulation [22–31], and others showing enhancement [30,32–42] (Table 1). Across these studies, electrical brain stimulation was applied with various ranges of parameters, with substantial differences in location, frequency, duration, amplitude, and timing (Fig 1). As well as technical challenges, a significant scientific challenge is that we do not yet have a complete characterization of the neural and electrophysiological correlates of memory. Researchers have identified a number of different electrophysiological signals that correlate with memory encoding, such as theta-band oscillations, which are potential targets for enhancement with stimulation. However, we do not know which electrophysiological signals are most directly and causally relevant for forming new memories. This uncertainty regarding the neural basis of human memory encoding makes it hard to precisely design stimulation protocols that are optimized to drive memory enhancement.

**Table 1:** Variability in stimulation protocols across memory modulation studies.

	Study	# of Subjects	Task	Neuronal changes	Region	Frequency	Amplitude	Pulse width	Duration	Task phase	Matter*
**IMPROVEMENT**	**[32]**	7	Spatial	Hippocampal theta phase resetting	Unilateral EC	50 Hz	0.5–1 mA	300 μs	5 s	Encoding	White matter
	**[33]**	11	Verbal	Rhinal-hippocampal gamma phase synchrony	Unilateral EC, Perirhinal, hippocampus	40 Hz	0.01 mA	Sine wave	400 ms	Throughout task	Gray matter
	**[34]**	11	Verbal	Increased hippocampus and posterior cingulate evoked responses	Fornix	5 Hz	8 mA	200 μs	4 h continuous	Before encoding	White matter
	**[35]**	4	Verbal, Complex figure, Naming	Evoked potentials in hippocampus	Fornix	200 Hz, theta burst 100 ms trains at 5 Hz	7 mA	100 μs	~1 h	Entire test	White matter
	**[36]**	102	Verbal	Increased spectral tilt	Mostly LTL	50 Hz	0.5–1.5 mA depth, 0.5–3 mA surface	300 μs	4.6 s	Encoding (closed-loop)	Both
	**[37]**	13	Person recognition	N/A	EC	Theta-burst microstimulation: 5 trains of 4 pulses at 100 Hz	150 uA	200 μs	1 s	Before encoding	Both (Effective: white matter)
	**[38]**	14	Item recognition	Increased theta-gamma phase-amplitude-coupling	Amygdala	Theta-burst stimulation: 8 trains of 4 pulses at 50 Hz	0.5 mA	500 μs	1 s	Encoding, image offset	Both
	**[39]**	22	Verbal free-recall	N/A	Lateral temporal cortex (no improvement when stimulating hippocampus, parahippocampal neocortex, prefrontal cortex)	50 Hz	0.5–3.5 mA	300 μs	4.6 s	Encoding	Both
	**[40]**	8	Delayed match to sample, delayed recall	Reproduced CA1 neuronal firing patterns during successful encoding	CA1	flexible	150 uA	1 ms	4 s	Encoding	N/A
	**[30]**	10	Verbal and associative	Increased theta power during retrieval	Hippocampus	50 Hz	2 mA	300 μs	5 s	Encoding	Anode in hippocampal gray matter; cathode in white matter
	**[42]**	22	Recognition and associative	N/A	Right EC	Macro: 50 Hz Micro: theta-burst: 5 trains of 4 pulses at 100 Hz	Macro: 0.4–6 mA Micro: 150 uA	Macro: 300 μs Micro: 200 μs	Micro: 1 s	Encoding	White matter
	**[41]**	18	Recognition memory	Enhanced sleep spindles, increased locking of neural spiking activity to MTL slow waves, and improved coupling between MTL ripples and thalamocortical oscillations	Orbitofrontal	100 Hz	0.5–1.5 mA	100 μs	50 ms	During sleep between consecutive day tasks	Both
**IMPAIRMENT**	**[22]**	4	Complex scene recognition	MTL excitation	Bilateral MTL (8–10 sites simultaneously)	Single pulse and pulse train	<1.5 mA	100 μs	100 μs or 500 ms	Encoding, Retrieval, Encoding + Retrieval	N/A
	**[23]**	12	Verbal	N/A	Unilateral hippocampal stimulation	60 Hz	.15–1.5 mA	1 ms	7 s on, 3 s off	Throughout task	N/A
	**[24]**	30	Verbal and visuospatial	N/A	Unilateral hippocampal stimulation	60 Hz	1.39–1.96 mA	1 ms	7 s on, 3 s off	Throughout task	N/A
	**[25]**	6	Recognition	N/A	Unilateral hippocampus	50 Hz	<2 mA	1 ms	1 ms	Encoding	N/A
	**[26]**	12	Recognition	No consistent response	Unilateral and Bilateral hippocampus	Monophasic single pulse	4–6 mA	1 ms	1 ms	Encoding, Retrieval, Encoding + Retrieval	Both
	**[27]**	49	Spatial and Verbal	No sustained phase reset	EC, hippocampus	50 Hz	0.5 mA–3 mA	300 μs	5 s (Spatial), 4.6 s (Verbal)	Encoding	Both
	**[28]**	5	Verbal	N/A	Left MTL	50 Hz	1.9–5.5 mA	300 μs	5 s	Between encoding and recall	N/A
	**[29]**	4	Spatiotemporal	Decoupling of spatial retrieval network	Broad cortical and subcortical network nodes	Theta-burst stimulation: 4 trains of 3 pulses at 50 Hz	4 mA	500 μs	2 s	Prior to retrieval cue	N/A
	**[31]**	10	Visual short term memory	N/A	MTL	50 Hz	3 mA	300 μs	1 s	Encoding, stimuli offset	N/A

*“Both” indicates if study tried gray- and white-matter targets even if the finding was about one type of matter.

One approach that can be employed is to stimulate in a way that would recreate or supplement the brain’s natural memory-related signals in order to improve the brain’s own natural memory encoding or retrieval state. In this approach, stimulation is often targeted at the hippocampus, a critical structure for memory. Individuals are stimulated with a fixed set of stimulation pulses designed to broadly excite hippocampal neural activity [23–27,30,33] or reinstate the hippocampus’ own endogenous 4- to 8-Hz theta rhythm. Because theta oscillations are linked to memory encoding and synaptic plasticity (Table 1) [37,38,42], researchers hypothesized that theta-enhancing stimulation would improve memory accuracy. Although this method seems logical, the effects on memory performance from hippocampal stimulation are mixed, perhaps reflecting our lack of understanding of how memories are encoded.

In this Essay, we discuss why the behavioral and physiological effects of invasive brain stimulation are so variable across studies and individuals, explaining how fixed approaches for memory enhancement produce inconsistent results. In this context, we explain why a more flexible approach that accommodates complex and heterogenous brain patterns of individuals is necessary for consistent memory improvement. Finally, we describe a framework for tailoring customized stimulation for memory enhancement based on each person’s individually mapped physiology, with a focus on the hippocampal–cortical network.

## Why are the effects of invasive brain stimulation so variable?

Given the importance of memory for everyday life, researchers have tried a number of approaches to apply electrical stimulation to enhance memory. Here, we focus on direct electrical stimulation with surgically implanted electrodes. Researchers have conducted studies with various types of stimulation, from continuous stimulation with direct currents to bursts of charge-balanced pulse trains with different parameters. Studies have also tested the effects of current applied at different times relative to behavior and across a range of target areas including both gray and white matter [37,42]. These different approaches and parameters have widely varying effects and need to be understood if we are to use invasive brain stimulation to effectively improve memory (Table 1).

### How does the precise location of stimulation impact memory?

Memory is a complex process that involves a broad network of brain regions. Many studies have attempted to improve human memory by applying stimulation to different elements of the memory network [54], with the aim of enhancing synaptic plasticity and memory formation; however, a wide range of behavioral effects have been observed.

In general, the behavioral effect of applying electrical stimulation to a region often corresponds with the broader functional role of that region (Fig 1A). For instance, stimulation in sensory regions induces perceptual phosphenes [55], the sensation of light without light actually entering the eye. However, the effects of stimulation even in a particular brain region can vary dramatically according to the specific positioning of the stimulating electrode(s). In some cases, stimulation at nearby sites within a region can produce highly variable, even opposite [56–59], behavioral effects. For instance, when direct electrical stimulation was applied to a specific cortical location, a patient spontaneously recalled memories from high school; however, when stimulation was applied at all neighboring locations it did not evoke high school-related memories [59]. To explain why the effects of invasive brain stimulation are so spatially specific, it is helpful to consider multi-scale interactions between the network and local physiology because stimulating different combinations of neuron populations and pathways can have complex network-level effects beyond the stimulation site [60].

The importance of the precise stimulation location is most evident from a set of recent memory-modulation studies that measured the positioning of individual stimulation electrodes with respect to white matter pathways (Fig 1A). White matter in the brain consists of bundles of axons that create structural pathways for communication between brain regions [61]. Due to the interconnection of regions by white matter tracts, stimulating nearby specific bundles can have particularly strong impacts. Two studies reported greater improvement in memory performance from stimulating in or close to the white matter pathways of the medial temporal lobe (MTL) rather than in gray matter (Table 1) [37,62]. This trend of different stimulation effects in white matter versus gray matter were also observed in the lateral temporal cortex [63]. These findings could be explained by the enhanced ability of white matter stimulation to modulate both local and distant neural activity [43,51,63,64], thus allowing for the regulation of broader networks that drive cognitive states [44–47,65,66]. Further, when stimulating gray matter directly, currents can impact neighboring cell bodies, and the effects are likely to be inhibitory and spatially limited compared to white matter [52,67,68]. Thus, to improve targeting of white matter with electrical stimulation, recent modeling work shows promise for selecting stimulation locations based on individual patient tractography [69,70].

Despite some apparent successes, merely stimulating the white matter elements of the memory network does not always lead to memory enhancement. For example, 2 studies [27,32] both targeted the white matter of the hippocampus and entorhinal cortex with 50-Hz stimulation and reported opposite effects on memory. This variability highlights the fact that effective stimulation for memory enhancement requires more than simply identifying a particular anatomical target. Therefore, even when applying stimulation in an optimal location, one may need to consider other aspects of the stimulation parameters with respect to task design, the type of memory process, and ongoing neural dynamics, because the wrong type of stimulation can be disruptive, even when applied even to the right target location (Table 1 and Fig 1B).

### Which stimulation parameters are most effective for memory modulation?

**Fig 2:**
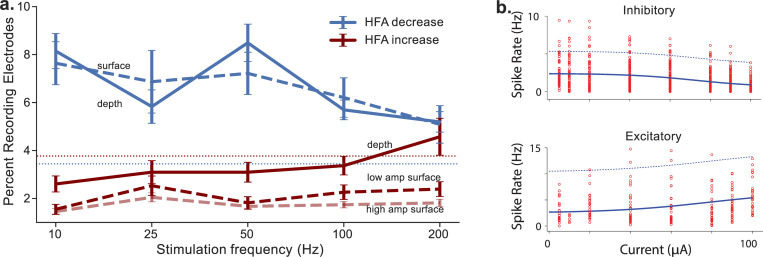
Percent of recording electrodes excited or inhibited by stimulation at different frequencies, amplitudes, and types of electrodes. (**a**) Percent of recording electrodes showing significant high frequency activity increases and decreases for each stimulation frequency for depth and surface stimulation sites. Adapted from [43]. (**b**) Increase in inhibitory and excitatory effects determined by spiking rate with stimulation amplitude. Adapted from [50].

Electrical stimulation can be applied to the brain with countless combinations of parameters, including different frequencies, amplitudes, and pulse-burst rates (Table 1 and Fig 1A). Identifying the optimal stimulation parameters is critical. The 2 stimulation parameters that often impact stimulation’s effects on underlying neural activity are the frequency and amplitude. Generally, applying low-frequency stimulation inhibits local neural activity, while high-frequency stimulation (100 Hz and above) can both excite or inhibit neural activity depending on the target region (Fig 2A) [43,51,71,72]. In contrast to frequency, where effects can be positive or negative, the effect of amplitude is generally simpler. At a given stimulation site, increasing amplitude increases the magnitude of the effect that occurred from lower-amplitude stimulation (Fig 2B) [51,64,72].

Beyond these general trends, there is additional variability across regions in the effect of stimulation frequencies that differ in relation to the functional role of the local circuit. In some brain regions, stimulation at particular frequencies can improve memory by up-regulating specific local processes. There is evidence of memory improvement from stimulation at higher frequencies [43], bursts of stimulation at 4- to 8-Hz theta frequencies [37,42,73], and lower amplitudes applied in deep structures. In particular, key hippocampal signals, such as ripple oscillations and 4- to 8-Hz theta rhythms, are vital for synaptic plasticity and memory in encoding, consolidation, and retrieval of information in spatial navigation, working memory, and long-term memory formation [74–77]. There is evidence that bursts of stimulation at theta frequencies can improve memory, likely by entraining and enhancing the brain’s own theta-band oscillations [78,79]. Meanwhile, 200-Hz stimulation in the temporal cortex has been used to target the spectral tilt in the power spectrum, which represents the prevalence of high- and low-frequency components in a neural signal. This biomarker, which is associated with memory-related increases in high-frequency activity, is thought to represent synaptic excitation/inhibition balance [80–83]. The ability of these contrasting approaches to elicit comparable memory enhancements demonstrates that there are multiple distinct neurophysiological signatures of memory processes that form effective stimulation targets [76]. An important challenge going forward is to identify the optimal neural signal to modulate in cases where there are multiple potential stimulation targets at different frequencies.

In addition to frequency and amplitude, the pulse width and waveform also are important factors in controlling how stimulation affects the brain (Fig 1A) [84,85]. Most memory modulation studies have applied charge-balanced, biphasic, rectangular pulses (Table 1). These rectangle-shaped voltages patterns, especially when delivered continuously for many seconds, are quite different from the brain’s native electrical patterns. Although research is limited, there is reason to think that alternative stimulation waveforms, such as sinusoids [67], may be helpful because the currents may more closely align with endogenous electrophysiology. An additional useful approach may be varying the width of stimulation pulses, as varying pulse widths may elicit the ability to recruit different types and sizes of neurons [86].

In recent years, researchers have identified a number of new, important electrophysiological patterns that may be useful targets for memory enhancement in humans, such as sharp-wave ripples and traveling waves of memory-related oscillations [87]. Up-regulating these signals with naturalistic stimulation may be more effective for memory improvement. Along these lines, one study experimented with stimulation that more closely resembled actual electrophysiological rhythms, with sine-wave-shaped stimulation delivered in-phase and anti-phase inducing memory enhancement and impairment, respectively [33]. A more recent study demonstrated impairment of memory retrieval by targeting separate nodes of the memory retrieval network with precisely timed theta-burst stimulation [29]. The specific phase lag that impaired memory the most varied depending on the stimulation target nodes of the network, emphasizing the importance of precisely timing stimulation to match the brain’s intrinsic signals. Thus, the efficacy of stimulation for cognitive enhancement may depend on finding the optimal combination of waveform shape, timing, frequency, amplitude, pulse width, and temporal patterning for a particular target brain region. Together, these studies open pathways for future work, such as designing stimulation waveforms to modulate endogenous neural signals and determining how to best deliver stimulation at multiple locations simultaneously.

### When should stimulation be applied for memory enhancement?

In order for stimulation to improve memory, it is not only important to choose the correct stimulation parameters and location, but also to find the right time to stimulate. The correct stimulation applied at the wrong time can be problematic; for instance, one study showed that stimulation applied in the period between encoding and recall impaired memory to a greater degree than when applied directly during either one of these 2 periods individually [28]. This amplified disruption implies an important functional process occurs between the encoding and retrieval phases of memory tasks (Fig 1B). Other research adds to this perspective showing that the effects of stimulation depend on the state of the memory network at the time of stimulation. One study showed that if a patient was already in a good memory encoding state, stimulation was not necessary [36]. However, if a patient was in a poor memory encoding state, stimulation could facilitate better memory performance [36]. Researchers have often assumed that stimulation during encoding would either disrupt or enhance neural mechanisms involved in memory formation, while stimulation during retrieval would modulate recall mechanisms (Table 1). However, studies applying stimulation between these explicitly defined encoding and retrieval periods indicate the importance of less understood dynamic memory processing during intermediary states.

Separate from wakeful encoding and retrieval memory states, one study modulated memory performance by stimulating during periods of sleep that occurred between task sessions [41]. Instead of aiming to induce a good memory encoding or retrieval state when a subject was trying to encode or retrieve information, this approach enhanced how newly encoded information was consolidated in order to be recognized more accurately the next day. Interestingly, even as stimulation was applied during unconscious sleep states, it changed neural activity in distinct ways from awake states [88]. The different effects of stimulation in wakefulness versus sleep call for further exploration of the underlying neural dynamics across diverse states to better predict large-scale responses to stimulation.

An additional challenge in designing brain stimulation protocols is that the same type of stimulation can have varying effects on different types of memory [30,38]. For instance, one case demonstrated that 50-Hz stimulation in the hippocampus improved associative memory even as it disrupted memory for individual items [30]. In line with this concept, a separate study demonstrated that multi-site stimulation targeting nodes of a network impaired spatial memory retrieval, but not temporal memory retrieval [29]. These findings further emphasize the importance of ensuring that stimulation is tailored according to current behavioral events and the brain’s ongoing functional processes.

## How do the effects of stimulation vary across individuals?

### Interindividual differences in anatomy and connectivity

Beyond parameter- and location-related effects, there are also substantial individual differences in the neural and behavioral effects of stimulation [56]. Individual humans have unique anatomy, connectivity, and functional organization, which influence how electrical stimulation interacts with local neural activity and brain circuits to modulate behavior. Early work [56,57] showed that when direct electrical stimulation was applied to similar locations on the surface of the sensory cortex, different patients experienced opposite sensations of involuntary vocalization or a temporary inability to speak, and in another case, numbness or feeling sensations without outside stimuli. These variations may be caused by various factors, such as differences in the exact location of neural populations, the density of neurotransmitter receptors, and the strength of synaptic connections. In addition, evidence from nonhuman primates suggests that specific patterns of stimulation can simultaneously induce both inhibitory and excitatory effects in different downstream regions [72]. A patient’s own individualized microarchitecture, or neural microstructure surrounding the stimulating electrodes, including the arrangement and density of cells, proximity to white matter tracts, functionally connected regions, and cortical layers, affect the way current flows through neural tissue and how neural activity changes in different downstream regions [42,43,45,51,52,63,89–93].

These diverse sources of variation create a challenge in designing brain stimulation protocols to alter brain activity in targeted ways that achieve desired electrophysiological changes and behavioral outcomes. Much of the apparent variability in therapeutic efficacy in treating neuropsychiatric disorders [21,94] likely stems from the largely uncharacterized and complex interactions between stimulation region, frequency, amplitude, pulse width, duration, and location relative to individual patients’ grey and white matter.

Researchers and clinicians should consider this individual- and region-level variability when crafting stimulation protocols. For instance, if the goal is to inhibit a specific brain region, one may design a stimulation protocol that targets gray matter with low-frequency stimulation; however, if the goal is to modulate large-scale connectivity, a stimulation protocol that targets a white-matter bundle in the relevant network with high-frequency pulses may be more effective. These optimal locations for each patient can be informed by mapping procedures, such as cortico-cortical evoked potential responses to single pulse electrical stimulation and structural connectivity from diffusion tensor imaging [51,63]. The neural and therapeutic impact of some of these interactions have been explored in animals and by using models [67–70], and in deep brain stimulation to treat Parkinson’s disease, providing a preliminary understanding of how combinations of stimulation parameters interact when influencing neural activity.

### Interindividual differences in memory-related brain oscillations

An additional factor that may modulate the effects of brain stimulation in a given region are the local electrophysiological characteristics of activity, in particular endogenous neural oscillations.

Key neural signals related to memory are neural oscillations in the theta and alpha band [95,96]. However, the frequencies of ongoing theta and alpha oscillations vary widely between subjects and across cortical regions [87,97]. In the human hippocampus, for example, theta- and alpha-like oscillations range from 2 to 16 Hz [98], with narrow-band theta oscillations at 4 to 8 Hz being associated with successful memory encoding, particularly when synchronized between regions. In addition, there is also evidence that “slow theta” oscillations at 3 Hz could relate to episodic memory [95]. A different memory-related pattern is high-gamma activity, often occurring at specific phases of low-frequency oscillations, which has also been associated with a wide range of memory and attention processes [82,99]. A specific type of high-frequency oscillation event, known as a ripple, has been shown to underlie the binding and retrieval of memories [100,101].

Across all these rhythmic brain signals there is substantial individual variability in the frequency of oscillations related to various factors, including genetics [102], age [103,104], ongoing cognitive process, and neurological disorders [105]. For instance, younger individuals tend to exhibit oscillations at faster frequencies in certain bands [106]. Thus, because brain stimulation interacts with network oscillations, these varying oscillation frequencies have implications for the effective use of stimulation for memory. As stimulation at different frequencies has distinct effects [48,107], researchers may tailor the frequency of stimulation to an individual’s own natural oscillatory frequencies. Some studies that used theta-burst stimulation have employed this approach to amplify a subject’s own theta oscillations [38,49,107]. However, it remains an open question if patients could benefit from receiving customized stimulation in relation to neural biomarkers at other non-theta frequencies, such as memory-related high-frequency activity [81,82].

Neural oscillations vary not only in their frequency between patients, but also in their spatiotemporal patterns across the brain. An emerging discovery is that many brain oscillations are not only local but instead they propagate across the cortex as traveling waves. Traveling waves of memory-related oscillations are present in many individuals. Moreover, traveling waves exhibit individual differences across subjects, including variations in the strength of propagation, frequency, and the direction of wave propagation [87,108,109]. These differences remain to be understood, but could arise from variability in cortical geometries between patients, which constrain the function of large-scale neural patterns [110]. Given that traveling waves may correlate with the propagation of task-related information across the brain, individual-level variations in the topographies and direction of traveling waves are likely to interact with the effects of stimulation. Therefore, stimulation may be more effective if protocols are designed to target characteristics, such as frequency and axis of propagation, of ongoing traveling waves.

## Conclusions and future directions

Although we have suggested useful future directions, there are several areas where more research is needed to create improved protocols for memory-related stimulation. The effects of stimulation on memory are diverse, with separate studies showing memory enhancement and disruption from stimulation. To explain this variability and help derive improved protocols, below we describe several general directions of future research that may be fruitful.

First, there should be more investigation into the sources of variability in human memory biomarkers and in effects of stimulation. Currently, one issue is that stimulating a fixed location with the same parameters in different patients or conditions leads to different neural and behavioral effects. We hypothesize that at least part of the heterogeneous effects of brain stimulation on human memory stem from uncontrolled variations in the brain’s anatomy, functional dynamics, pathology, and instantaneous state. We need to advance our measurements of these factors and then take them into account to guide stimulation location and parameters.

To advance towards a personalized stimulation framework for memory, we should, in particular, probe variability in the hippocampal-cortical network. The individual differences from the effects of stimulation can be substantial (with some subjects often showing effects in opposite directions [43]). To explain this variability, we should probe all possible sources, such as microarchitecture differences related to cell density or tractography, as well as macro-level features related to neural anatomy, memory biomarkers, and brain connectivity. If we have an improved sense of this variability in the hippocampal-cortical network it could lead to improved subject-specific stimulation frameworks for memory.

Further, it may be useful to identify factors that predict the effects of stimulation in individuals, perhaps by using genetic markers that influence neurotransmitter levels, characteristics of neural activity, and neural plasticity in combination with or novel neuroimaging [111]. This approach could identify patients who are most likely to benefit from optimization of stimulation therapy. Personalization of stimulation protocols could go further, including patient-specific customization based on the task, and phase of memory processing, frequency of neural oscillations, and adjusting stimulation parameters accordingly such that stimulation is applied in a real-time, closed-loop fashion [36,53,112].

Currently, much of our understanding of human brain function is derived from animal studies. However, for the purpose of creating human brain stimulation protocols to modulate memory, it seems that animal studies have limited utility because there seem to be key behavioral and electrophysiological differences in terms of how complex memory processes operates in humans. As one example, theta-band rhythms often appear robustly in rodents at 8 Hz, whereas these signals are slower and transient in humans [74]. Given these differences in such a key memory-related signal, we need continued research to understand the aspects in which brain stimulation in the human hippocampus can rely on rodent models versus the circumstances in which we need human-specific data and models.

An additional challenge going forward is to evaluate the degree to which the neuromodulation paradigms developed in the clinic translate to the real world. Most of the study paradigms that form the basis of our current understanding of human brain stimulation for memory are based on memory tasks running on laptop computers at a patient’s bedside. Memory in the real world, with distractions and many implicit tasks, may utilize different neural processes from computer-based paradigms. Thus, acute memory modulation in a clinical setting may not always be indicative of sustained improvements in real-world memory function. It will take creative unification of multiple research areas going forward to identify the aspects of brain stimulation research and paradigms that are most useful for memory enhancement in naturalistic settings.
